# Validation of a General and Sport Nutrition Knowledge Questionnaire in Adolescents and Young Adults: GeSNK

**DOI:** 10.3390/nu9050439

**Published:** 2017-04-29

**Authors:** Patrizia Calella, Vittorio Maria Iacullo, Giuliana Valerio

**Affiliations:** 1 Department of Movement Sciences and Wellbeing, Parthenope University, Naples 80133, Italy; giuliana.valerio@uniparthenope.it; 2 Department of Psychology, Sapienza University of Rome, Rome 00185, Italy; vittoriomaria.iacullo@uniroma1.it

**Keywords:** nutritional knowledge, questionnaire, sports, adolescents, young adults

## Abstract

Good knowledge of nutrition is widely thought to be an important aspect to maintaining a balanced and healthy diet. The aim of this study was to develop and validate a new reliable tool to measure the general and the sport nutrition knowledge (GeSNK) in people who used to practice sports at different levels. The development of (GeSNK) was carried out in six phases as follows: (1) item development and selection by a panel of experts; (2) pilot study in order to assess item difficulty and item discrimination; (3) measurement of the internal consistency; (4) reliability assessment with a 2-week test-retest analysis; (5) concurrent validity was tested by administering the questionnaire along with other two similar tools; (6) construct validity by administering the questionnaire to three groups of young adults with different general nutrition and sport nutrition knowledge. The final questionnaire, consisted of 62 items of the original 183 questions. It is a consistent, valid, and suitable instrument that can be applied over time, making it a promising tool to look at the relationship between nutrition knowledge, demographic characteristics, and dietary behavior in adolescents and young adults.

## 1. Introduction

Adolescence is a period of rapid growth and changes; therefore, good food habits and adequate nutrient intake are crucial to ensuring optimal development during this life stage. Many fields of society such as schools, families, sports coaches, and the mass media play a critical role in influencing the dietary behaviors of adolescents and young adults. A well planned nutrition program is also important to obtain and maintain good athletic performance [1]. However, recreational and competitive young athletes frequently have suboptimal or unbalanced dietary intakes, despite having their attention turned to physical fitness [2]. Food intake is important not only in terms of the energy content of ingested food, but also in terms of the quality of the diet. Sport settings frequently offer direct access to high energy and/or low nutrient snacks and beverages, such as candy bars, cookies, chips, protein bars, sports or energy drinks, and soft drinks [3]. Youth sports marketing strategies may actually encourage associating these products with health and fitness activities [4]. In addition, time pressures derived from the sport practices and events may lead to heightened consumption of fast food, which are very likely unhealthy [5]. Furthermore, adolescent athletes have no knowledge or perception whatsoever of their nutrition status, so the use of supplements is not driven by an awareness of insufficiency, but instead a desire for performance enhancement [6]. 

Good nutrition knowledge is one of the few modifiable determinants of dietary behaviors and contributes to strengthening the skills and abilities needed to resist the environmental influences leading to unbalanced and unhealthy diets [7]. For these reasons, the focal point of the assessment of general and sport nutritional knowledge is to raise the awareness of good dietary habits and to improve appropriate nutritional knowledge, thereby helping to dispel myths and false beliefs in young athletes.

A very recent systematic review by Trakman et al. [8] focused on nutrition knowledge questionnaires for athletes and coaches and highlighted the low quality of these tools. The authors concluded that none of the tools analyzed could be confidently endorsed for use in future studies. In fact, most questionnaires did not consider health literacy and lacked appropriate and adequate validation [9,10]. This limitation may explain the reason why the studies investigating the relationship between nutrition knowledge and dietary behavior in athletes and coaches failed to find any significant associations [2,9]. 

As far as the authors are aware, no psychometrically validated questionnaires exploring both general and sport nutrition knowledge are available for the Italian population. 

The aim of this study was to develop a new tool to measure the general and the sport nutrition knowledge of adolescents and young adults. Following a rigorous validation procedure, the final questionnaire would be a useful instrument for researchers and professionals to evaluate the knowledge and knowledge gaps in general and sport nutrition, to evaluate the relationship between knowledge and behavior, to assess the effect of nutrition educational programs, and to as a useful instrument to test the water in order to build targeted educational programs.

## 2. Methods and Results

**Figure 1:**
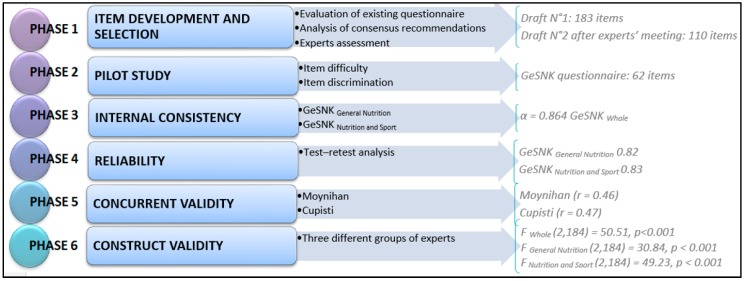
Flow chart of the development and validation of the general and sport nutrition knowledge (GeSNK) questionnaire.

We based methods and procedure on the general guidelines of the item response theory framework [9] used to validate reliable questionnaires. Firstly, we developed and selected the items, using an experts panel, then we did a pilot study to ‘sharpen’ the tool using the item difficulty index and the item discrimination index. Lastly, we validated the questionnaire on a large sample computing internal validity, concurrent validity, internal consistency, construct validity, and reliability. Hereafter for each validation phase, the method and results will be displayed to make the procedure easily understandable. Figure 1 shows the whole procedure followed for the development and validation of the general and sport nutrition knowledge questionnaire.

### 2.1. Phase One—Item Development and Selection

Considering that nutritional recommendations may differ between athletes and the general population, we decided to divide the questionnaire into two main sections. The first section concerns general nutrition; the aim of this section is to explore knowledge about the predominance of specific macronutrients or micronutrients in various foods, practical food choices, and awareness of diet-health associations. The second section is instead specific for sports nutrition; in this section, we aimed to explore knowledge about fluid replacement, supplement intake, and food and timing choices in recovery meals. A preliminary item pool was generated from an expert panel composed of a pediatrician and a dietitian with expertise in nutrition and sport. They produced the items according to the recommendations and guidelines [1,11,12]. The panel was successively extended to another pediatrician, another dietitian, and two psychologists that selected the best items in terms of interpretability, clarity, importance of their contents, and pertinence.

#### Phase One—Results

The preliminary item pool was composed of 183 questions which we had developed and formulated. Firstly, each member of the extended expert panel received the questionnaire by e-mail and reviewed the items using the guidelines reported in Appendix A. Seventy-three items did not reach the minimum requirement of clarity, interpretability, importance of their contents, and pertinence. At the end of this first qualitative review, the second round was performed in a face-to-face meeting, where the extended expert panel reviewed the whole questionnaire, and discussed and approved the pilot version of the questionnaire, which was composed of 110 items. 

### 2.2. Phase Two—Pilot Study: Item Difficulty and Item Discrimination

We submitted the preliminary version of the questionnaire to 269 high-school students in order to assess item difficulty and item discrimination. Participants were students of “Liceo Assteas”, in Buccino (Salerno), Italy. The students completed the questionnaire during their class schedule. The main investigator (CP) administered the questionnaire under the supervision of the teacher and provided oral instructions about its completion. The importance of answering the questions honestly was explained. The students were allowed to write any pertinent comments for each item in order to provide qualitative feedback. Moreover, for each class, we had a debriefing session once all the students had finished, in which they could bring up additional comments and suggestions.

#### Phase Two—Results

The performance of 19 participants was not considered because the investigators judged that they did not complete the questionnaire in a serious manner. Thus, the total sample was composed of 250 students (104 males, 146 females, age = 16.63 ± 1.57 years).

Item difficulty: the difficulty index of the 110 item questionnaire was calculated by dividing the number of high school students who correctly answered each item by the total number of the students who answered it [13], according to the following formula:

$$p_{i}=\frac{A_{i}}{N_{i}}$$

where:

*p_i_* = Difficulty index of item *i*
*A_i_* = Number of correct answers to item *i*
*N_i_* = Sum of correct plus incorrect answers to item *i*

The greater the difficulty of an item, the lower the index. According to Kline [14], items that were too simple or too difficult were rejected. Thus, all the items with a difficulty index over 0.80 or under 0.20 were rejected. Moreover, we eliminated all the items containing technical words that were judged incomprehensible even by a minor part of the subjects (e.g., bigorexia, prebiotic, and probiotic).

Item discrimination: we measured the ability of each item to discriminate between high-school students with different knowledge levels by correlating the score on each item with the overall test score. A correlation lower than 0.2 was considered enough to discard an item [15], unless the item was considered essential in terms of content validity. At the end, none of the items reported a negative correlation, while 48 items reported a correlation lower than 0.2 and were excluded. None of the excluded items tested an essential aspect of nutrition knowledge not yet covered elsewhere in the questionnaire. The final questionnaire, consisted of 62 items (hereafter GeSNK).

### 2.3. Phase Three—Internal Consistency 

The internal consistency, assessing whether several items referred to the same general construct producing similar scores, was measured separately for the two different sections of the GeSNK questionnaire:

- GeSNK General Nutrition
- GeSNK Nutrition and Sport

For this purpose, the GeSNK questionnaire was administered to a validation sample of 202 high-school students, attending first to fifth period classes at the “Liceo Sannazaro” (*n* = 92) and “Liceo Mazzini” (*n* = 110) in Naples, Southern Italy.

In this phase, we collected some additional information: gender, date of birth, height and weight, sport participation and weekly frequency and duration, parents’ level of education, and parents’ employment status. The physical activity levels were defined in two categories: active youth, who practiced a sport at least two times in a week; and inactive youth, who did not practice any kind of physical activity. Three levels of parental education were considered: degree, high-school leaving certificate, and lower qualification. Three levels of parental employment were considered, Level I: businessman, manager, professional; level II: office worker; level III: manual worker, craftsman, homemaker [16]. Table 1 shows the characteristics of the validation sample.

The administration procedure was the same as in the pilot study. The internal consistency for each section was calculated using the Cronbach’s alpha: Kline [14] recommended a score of 0.7 as the minimum requirement for internal consistency.

#### Phase Three—Results

The Cronbach’s alpha was: 0.84 for GeSNK General Nutrition and 0.71 for GeSNK Nutrition and Sport. The total internal consistency, computed by calculating the sum of the two sections of the GeSNK (GeSNK Whole), was α = 0.86. With regard to the influences of the parents’ socioeconomic status (job and the level of education), no significant differences were found between the nutritional knowledge of the adolescents. This result shows that nutritional knowledge is not influenced by the social-economic variables considered. 

### 2.4. Phase Four—Test-Retest Reliability 

To measure the reliability of the GeSNK questionnaire, a 2-week test-retest analysis was conducted in the validation sample. The students were asked to complete the questionnaire again 2 weeks later, under the same conditions. The 2-week period is considered an acceptable length of time for participants to exclude learning and memory effects [14]. The responses from the first and the second time that the questionnaires were completed were matched using ID numbers. Test-retest reliability was assessed by using Pearson’s correlation to demonstrate that the results were consistent over time [17].

From the initial sample of 202 high school students, only 137 (67.8%) completed the GeSNK questionnaire twice (see Table 1). Thus, 65 participants missed the retest phase, because they were absent on the retest day. The characteristics of the 137 students participating to the test-retest analysis did not differ from the initial sample.

#### Phase four—Results

The reliability for the GeNSK General Nutrition was 0.82, for the GeNSK Nutrition and Sport the reliability was 0.83, and for the GeNSK Whole the reliability was 0.85. According with Nunnally (1978) a satisfactory level of reliability depends on how a measure is being used; we consider a coefficient > 0.70 satisfactory for our questionnaire [17]. Our decision is made not on the metropolitan legend of *r* > 0.70 [18], but on the fact that these coefficients are as high as those reported by other studies [15,19,20,21,22].

### 2.5. Phase Five—Concurrent Validity

To test the concurrent validity, the GeSNK questionnaire was administered on the same sample with two more similar questionnaires. From an extensive literature research, we found two questionnaires tested on Italian subjects that were suitable for comparison, namely the questionnaire proposed by Moynihan et al. [23], which assessed general nutrition knowledge, and the questionnaire proposed by Cupisti et al. [24], which assessed nutrition knowledge in athletes. The GeSNK questionnaire, as well as Moynihan’s and Cupisti’s questionnaires were administered to the validation sample at the beginning of the study.

The administration protocol was the same as in the pilot study; the three questionnaires were administered in the same day.

#### Phase Five—Results

Table 2 shows correlations among Moynihan’s or Cupisti’s questionnaires and GeSNK General Nutrition, GeSNK Nutrition and Sport, or the GeSNK Whole. Higher correlation coefficients were found between Moynihan’s and GeSNK General Nutrition than Cupisti’s and GeSNK Nutrition and Sport. Moreover, both questionnaires showed a fair correlation close to 0.50 with the GeSNK Whole. The correlation between the Moynihan’s or Cupisti’s questionnaires were 0.433 (*p* < 0.001). Since the construct of nutrition knowledge is very broad and each tool considered here measures it from a different point of view we observed a not very high concurrent validity.

It is unlikely that the GeSNK has a low precision with respect to the other two questionnaires, otherwise we should have observed a high correlation between the other two questionnaire and low correlations between the GeSNK and each of them. The results tell something different. The correlation between the other two questionnaires is 0.433, comparable with the correlations between the general score of the GeSNK and each of them (0.456 and 0.470).

### 2.6. Phase Six—Construct Validity

The final step was to assess the construct validity by administering the GeSNK questionnaire to three groups of young adults, who were expected to differ in their nutrition and sport nutrition knowledge. This step was performed only in adults due to the unfeasibility of discriminating between experts and non-experts among the adolescents.

The sample was composed of undergraduate and postgraduate students in Dietetics (enrolled at the Federico II University of Naples) and Movement Science (enrolled at the Parthenope University of Naples), who were supposed to be knowledgeable in general nutrition and/or sport nutrition, and undergraduate students and postgraduates in Economics (enrolled at the Parthenope University), as a control group. The mechanism for recruitment was visiting the classrooms of consenting professors, at the end of a lecture at the university. Participants were eligible if they were between 18 and 32 years of age. One-hundred-eighty-eight participants were enrolled and the following categories were represented: 49 Dietitians or students of Dietetics, 83 Sport Scientists or Movement Science students, and 56 Economists or students of Economics (see Table 3). 

As in the adolescent sample, the following additional information was added to the GeSNK questionnaire: gender, date of birth, height and weight, sport participation and weekly frequency and duration, main occupation, and education level.

#### Phase Six—Results

Both the Dietetics and Nutrition and the Movement Science groups scored consistently higher than the Economics group on both sections of the GeSNK (*p* < 0.001). A 3 × 3 ANOVA reported significant main effects for the general nutrition knowledge factor (*F* (2, 184) = 30.84, *p* < 0.001), the sport and nutrition knowledge factor (*F* (2, 184) = 49.23, *p* < 0.001), and the total score (*F* (2, 184) = 50.51, *p* < 0.001).

**Figure 2:**
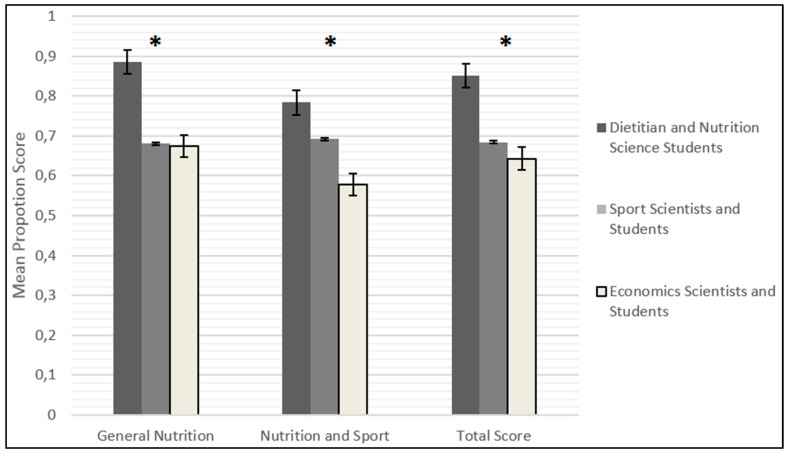
Difference of the mean score of the GeSNK questionnaire among the two groups of experts (Dietitian and Nutrition Science students; Sport Scientists and students) and the control group (Economics Scientists and students). Note: * *p* < 0.001.

The Tukey honest significant difference test showed that the Dietetics and Nutrition students obtained the highest mean score in the two sections as well as the total score of the GeSNK. Despite the score of the Movement Science group not differing significantly from the Economics group in the general nutrition knowledge factor, the score of the Movement Science group was significantly higher in comparison to the score of the Economics group for sports and nutrition knowledge section (*p* < 0.001). This result is consistent with the hypothesis that the GeSNK sports nutrition section effectively measures the nutrition knowledge applied to sport (see Figure 2). The questionnaire therefore met the criterion for construct validity.

### 2.7. Statistics

Differences between knowledge scores (total and sub-scores on the two sections of the instrument) were assessed using one-way ANOVA.

Internal consistency of the instrument was measured using Cronbach-α constructs, which evaluate how consistently items within each section of the instrument and overall score assess the knowledge. Cronbach-α values range from 0 to 1, with this scale indicating the consistency of responses. Pearson’s correlation was used to assess the test-retest reliability of the GeSNK. A score of 0.7 or greater is considered satisfactory both for internal consistency and reliability [13,14]. Concurrent validity was also computed using Pearson’s correlation between the GeSNK and the two other questionnaires. The construct validity, instead, was evaluated considering significant differences between two groups of experts (in nutrition and sport sciences) and a control group, using one way ANOVA. Significance was set at *p* = 0.05. Data were analyzed using the Statistical Package of Social Sciences (SPSS, Chicago, IL, USA) for Windows software program (version 17.0). 

### 2.8. Score

The normative data are based on the performance of the test phase of the validation sample. Thus, without considering data of the retest phase provided by the same sample. The majority of the questions could be answered ‘True’, ‘False’, or ‘I don’t know’. The first eight questions were about the main macronutrient and micronutrient contents in some food and could be answered ‘High’, ‘Low or Absent’, or ‘I don’t know’. Scores were coded as + 1 for a correct answer, and 0 if participants selected the incorrect answer or the ‘I don’t know’ response. The GeSNK questionnaire included 62 items. The general nutrition section included 29 items, whereas nutrition and sport section included 33 items. The maximum total score was 97 and the minimum was 0. For the general nutrition section, the maximum score was 64, for the nutrition and sport section the maximum score was 33.

**Figure 3:**
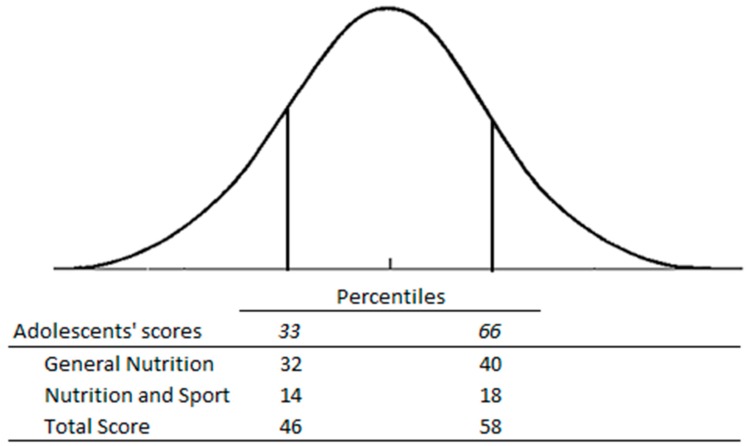
Scores that define three scores categories: low, medium, and high knowledge level in adolescents.

When the GeSNK is submitted to adolescents, the knowledge of participants that obtain a score lower than the 33rd percentile (32 in general nutrition, 14 in nutrition and sport, 46 in total score) is considered as low. A score included between the 33rd and 66th percentile is considered medium. Performance higher than the 66th percentile (40 in general nutrition, 18 in nutrition, and sport, 58 in total score) is labelled as high knowledge (see Figure 3). Normative data for the adult sample should be collected in the future.

### 2.9. Ethics

The Ethics Committee of the University of Naples Federico II approved the study protocol (ref. 28/16) and written informed consent was obtained from the adolescents and parents involved in the pilot and validation samples, and from the young adults.

## 3. Discussion

The present study aimed to develop a new questionnaire exploring general nutrition and sports nutrition knowledge using a mixed sample of adolescents and young adults doing sports at different levels (from sedentary to athletes). The original version of the questionnaire is in Italian, but because we did not mention any typical Italian dishes or content related exclusively to the Italian tradition that are not in use in other countries, it is usable in other countries in case of lack of validated questionnaire.

Despite the complexity of the relationship between nutrition knowledge and dietary intake, stronger associations might be identified using an instrument that is able to accurately measure nutrition knowledge and more precisely assesses dietary intake [21].

As Trakman et al. [8] maintained, many studies aiming to assess nutrition knowledge in athletes lacked appropriate and adequate validation procedures. In a recent review, they analyzed 36 studies and none of them used a questionnaire that covered the whole nutrition sub-sections (*n* = 11) that were deemed relevant (general nutrition knowledge, protein, carbohydrate, fat, micronutrients, nutrition pre/during competition, recovery meal, fluid, supplements, and alcohol). The GeSNK questionnaire covered 10 of these sub-sections with the exception of the knowledge about alcohol, which we considered not relevant to assess the knowledge in nutrition. In their review, they explored how many studies considered the pre-test analysis, the face validity, the content validity, the item discrimination, the internal reliability, the construct validity, and the external reliability. The GeSNK questionnaire validation process covered all these psychometric requirements, also testing the concurrent validity using two other different tools.

The pilot study is a preliminary study conducted to test aspects of the tool design and to allow necessary adjustment before final commitment to the questionnaire. In many studies a pilot study was not performed [25,26,27,28]. All these studies did not report items’ difficulties, which excludes the possibility of having items too easy or too difficult to perform removed from final questionnaires. The pilot study of the GeSNK questionnaire was performed on a large sample with a range of 14 to 19 years of age, which differed from two previous studies that used a smaller sample [29,30]. To develop our GeSNK questionnaire, the expert panel reviewed the pilot questionnaire to establish face validity and content validity. Other studies tested only one of these two validity indexes [30,31,32]. The latter procedure assesses whether the items in the instrument are appropriate and can comprehensively measure all the aspects of the construct being considered. In the present study, we measured the ability of each item to discriminate between high-school students with different knowledge levels by correlating the score of each item with the overall test score.

Internal consistency is an essential step for the psychometric validation. We calculated it for the entire questionnaire and for the two sub-sections of the GeSNK, achieving good results.

Construct validity is established if the instrument is able to identify differences in scores between groups; different studies examined it using only two different groups. We selected three different groups with different skills: significant differences between the scores of the Dietitians and Nutrition group and Economics group (control group) were reported in the GeSNK General Nutrition. Despite the score of the Movement Science group not differing significantly from the Economics group for the general nutrition, they reported a significantly higher score in the GeSNK Nutrition and Sport section. This finding indicates that the questionnaire is able to differentiate two different aspects of nutrition knowledge for whom it was created: general nutrition and nutrition related to sport. Moreover, we report that the dietetic students scored higher on all sections of the questionnaire.

External reliability refers to the extent to which a measure varies from one use to another. External reliability can be assessed using the test-retest method. The satisfactory performance (*r* ≈ 0.70) obtained after two weeks of test-retest would suggest that the GeSNK had good external reliability.

Furthermore, the GeSNK questionnaire was correlated with two more nutritional knowledge questionnaires [23,24] to assess the concurrent validity. This seems to be rare in this field, computing this construct falls in line with what Trackman et al. [8] proposed. Concurrent validity is a measure of how well a particular test correlates with a previously validated measure and contributes to estimating the precision of the tool. If two tools measure the same construct then they are expected to correlate positively. All the questionnaires correlated positively around 0.50, meaning that they measure the same construct. However, since these correlations are not very high, we can assume that they do it from different points of view. Moreover, the Nutrition and Sport section of the GeSNK weakly correlated with the score of the other two questionnaires. The main reason of this result is due to the specificity of this section with respect to the total score of the other two questionnaires. In fact, the construct validity demonstrated that this section of the GeSNK effectively measures the nutrition knowledge related to sport, something very specific with respect to the general nutrition knowledge measured by the other two questionnaires used to compute the concurrent validity.

Considering the validation and reliability results of the GeSNK questionnaire, it can be proposed as a new, up-to-date measure of general and sports nutrition knowledge. Moreover, the questionnaire has the capacity to report a “knowledge profile”, outlining areas where knowledge is well understood and also gaps in knowledge. By “knowledge profile” we mean the possibility to classify the participants according with their total score and the score obtained in the General Nutrition section and Sport and Nutrition section: a score lower than the 33rd percentile is considered a “low profile of knowledge”. A score included between the 33rd and 66th percentile is considered a “medium profile of knowledge”. Lastly a score higher than the 66th percentile can be considered a “high profile of knowledge”. Finally, we report no relationships between nutrition knowledge and sport activity. However, our study was not designed to investigate this hypothesis, but just to validate a new tool. 

## 4. Conclusions

The GESNK questionnaire can be used in future studies to look at the relationships between nutrition knowledge, demographic characteristics, and dietary behavior, both in adolescents and young adults in the general population and in athletes. Moreover, future research studies could use this questionnaire to investigate the relationship between nutrition knowledge and sport activity, and also the differences in nutrition knowledge for different sports and sport levels. For this purpose, the section of Nutrition and Sport of the GeSNK would be particularly useful.

This could also represent a valid instrument for coaches and dietitians to rapidly evaluate gaps in nutrition knowledge. Furthermore, due its reliability, it could be a valuable assessment tool in planning nutrition educational programs and evaluating their efficacy.

## Figures and Tables

**Table 1:** Validation sample (*n* = 202) and test-retest sample (*n* = 137) characteristics.

		Validation Sample		Test-Retest Sample	
		*n*	%	*n*	%
Gender					
	Male	86	42.6	63	46.0
	Female	116	57.4	74	54.0
Age					
	<16	84	41.6	56	40.9
	16–17	87	43.1	61	44.5
	>17	31	15.3	20	14.6
Father job					
	Missing	31	15.3	21	15.3
	Businessman, Manager, Professional	78	38.6	51	37.2
	Office worker	76	37.6	54	39.4
	Manual worker, Homemaker	17	8.4	11	8.0
Mother job					
	Missing	79	39.1	52	38.0
	Businessman, Manager, Professional	31	15.3	23	16.8
	Office worker	62	30.7	43	31.4
	Manual worker, Homemaker	30	14.9	19	13.9
Father level of education					
	Missing	33	16.3	21	15.3
	Degree	107	53.0	72	52.6
	High school	58	28.7	40	29.2
	Lower qualification	4	2.0	4	2.9
Mother level of education					
	Missing	75	37.1	52	38.0
	Degree	75	37.1	23	16.8
	High school	42	20.8	43	31.4
	Lower qualification	10	5.0	19	13.9
Sport					
	Inactive people	87	43.1	42	30.7
	Active people	115	56.9	95	69.3

**Table 2:** Correlations of the GeSNK questionnaire with two questionnaires of nutritional knowledge (Moynihan et al. 2007; Cupisti et al. 2002).

	GeSNK Questionnaire		
*n* = 202	GeSNK _General Nutrition_	GeSNK _Nutrition and Sport_	GeSNK _Whole_
Nutritional knowledge questionnaire by Moynihan et al. [23]	0.452 *	0.277 *	0.456 *
Nutritional knowledge questionnaire by Cupisti et al. [24]	0.436 *	0.344 *	0.470 *

Note: * *p* < 0.001.

**Table 3:** Sample characteristics (*n* = 188).

	*n*	%
Gender		
Male	79	42.0
Female	109	58.0
Age		
18–20	74	39.8
21–23	43	23.1
24–26	34	18.3
27–30	18	9.7
30–32	17	9.2
Sport		
Inactive people	48	25.5
Active people	140	74.5
Degree		
Undergraduates	115	61.2
Postgraduates	73	38.8

## Supplementary Materials

The following are available online at http://www.mdpi.com/2072-6643/9/5/439/s1.

## Appendix A

Here we report the guidelines used by the experts to determine the clarity, interpretability, pertinence, and importance of each item.

Please for each item provide a score from 0 to 10. You have to assess its clarity, interpretability, importance, and pertinence with the content of the questionnaire. Below is the definition for each attribute. Please express your score according to the following definitions:

- Clarity: it refers to the language used. How appropriate and easy to understand is the language used?
- Interpretability: considering the contents and the terms used, how understandable is each item?
- Importance: please say, from your professional point of view, how important it is to include each item
- Pertinence with the content of the questionnaire: does the content of the item relate with general nutrition and sport?
