# Supplementary material for: Validation of a General and Sport Nutrition Knowledge Questionnaire in Adolescents and Young Adults: GeSNK
Source: Nutrients. 2017 Apr 29;9(5):439. doi: 10.3390/nu9050439 (PMC5452169; doi:10.3390/nu9050439)
Supplement: Supplementary file 1 [file nutrients-09-00439-s001.pdf]

GeNSK- General and Sport Nutrition Knowledge Questionnaire

The purpose of this questionnaire is to identify myths and false nutritional beliefs in order to improve nutritional education programs

Name/Surname:
Gender: Male ☐ Female ☐
Date of Birth:
Weight: Height:
Employment father/mother:

Level of education father/mother:
Do you practice any Sport? YES☐ NO☐
Sport:
How many times a week:
Average length: *(minutes per day)***:**

My knowledge about healthy eating are driven by: (you can choose more than one answer)

Nutritional education programmes at school	☐	*From that i see on the TV*	☐
Nutritional education programmes in other places	☐	*From the web*	☐
From what my teachers tell me	☐	*From my friends*	☐
From what my parents tell me	☐	*I have no knowledge about to healthy eating*	☐
From what my coaches tell me	☐	*Anything else……………………………………………………*	

SECTION 1: GENERAL NUTRITION

Following are the questions refer to the nutritional composition of some foods. Choose the answer with an X.

1. **The Carbohydrate content of such foods is:**

	High	Low or absent	I do not know
Boiled ham	☐	☐	☐
White bread	☐	☐	☐
Tomato	☐	☐	☐
Apple	☐	☐	☐
Ricotta	☐	☐	☐
Breackfast cereals	☐	☐	☐

2. **The Protein content of such foods is:**

	High	Low or absent	I do not know
Chicken meat	☐	☐	☐
Dried beans	☐	☐	☐
Pear	☐	☐	☐
Rice	☐	☐	☐
Codfish	☐	☐	☐
Parmesan	☐	☐	☐
Chocolate	☐	☐	☐

3. **The Fat content of such foods is:**

	High	Low or absent	I do not know
Salami	☐	☐	☐
Mayonnaise	☐	☐	☐
Dried chickpeas	☐	☐	☐
Pasta	☐	☐	☐
Butter	☐	☐	☐
Jam	☐	☐	☐

4. **The Fiber content of such foods is:**

	High	Low or absent	I do not know
Honey	☐	☐	☐
Brown bread	☐	☐	☐
Chicken broth	☐	☐	☐
Potatoes	☐	☐	☐
Pear	☐	☐	☐
White bread	☐	☐	☐

5. The <u>Salt</u> content of such foods is:

	High	Low or absent	I do not know
White bread	☐	☐	☐
Courgettes	☐	☐	☐
Canned peas	☐	☐	☐
Canned tuna	☐	☐	☐
Frozen peas	☐	☐	☐

6. The <u>Calcium</u> content of such food is:

	High	Low or absent	I do not know
Fesa di tacchinoTurkey	☐	☐	☐
Peas	☐	☐	☐
Walnut	☐	☐	☐
Olive oil	☐	☐	☐
Brown bread	☐	☐	☐

7. The <u>Iron</u> content of such foods is:

	High	Low or absent	I do not know
Calf meat	☐	☐	☐
Apple	☐	☐	☐
Honey	☐	☐	☐
Sea bass	☐	☐	☐

8. The <u>Potassium</u> content of such foods is:

	High	Low or absent	I do not know
Pasta	☐	☐	☐
Dried lentils	☐	☐	☐
Olive oil	☐	☐	☐
Honey	☐	☐	☐

Are these claims true or false? (only one answer is possible)

**9**	*The egg white is high in cholesterol*		
	**TRUE** ☐	**FALSE** ☐	**I DO NOT KNOW** ☐
**10**	*The high fat meals are ever high in cholesterol*		
	**TRUE** ☐	**FALSE** ☐	**I DO NOT KNOW** ☐
**11**	*The olive oil is high in monounsaturated fat*		
	**TRUE** ☐	**FALSE** ☐	**I DO NOT KNOW** ☐
**12**	*The dried fruit is a good source of essential fatty acids*		
	**TRUE** ☐	**FALSE** ☐	**I DO NOT KNOW** ☐
**13**	*The repened cheese are saltier than the fresh one*		
	**TRUE** ☐	**FALSE** ☐	**I DO NOT KNOW** ☐
**14**	*An high-energy food is exclusively a fat food*		
	**TRUE** ☐	**FALSE** ☐	**I DO NOT KNOW** ☐
**15**	*The brown bread is richer in fiber than the white one*		
	**TRUE** ☐	**FALSE** ☐	**I DO NOT KNOW** ☐
**16**	*Bran is the outer part of the grain kernel that is very high in fiber*		
	**TRUE** ☐	**FALSE** ☐	**I DO NOT KNOW** ☐
**17**	*Tinned pulses are saltier than the dry one*		
	**TRUE** ☐	**FALSE** ☐	**I DO NOT KNOW** ☐
**18**	*Omega-3 and omega-6 are particular fatty acids*		
	**TRUE** ☐	**FALSE** ☐	**I DO NOT KNOW** ☐
**19**	*Our body creates vitamin D from direct sunlight on our skin when we are outdoors*		
	**TRUE** ☐	**FALSE** ☐	**I DO NOT KNOW** ☐
**20**	*The iron in meat are more easily absorbed than the same mineral found in vegetables*		
	**TRUE** ☐	**FALSE** ☐	**I DO NOT KNOW** ☐
**21**	*A variety of foods contain a natural amount of sodium*		
	**TRUE** ☐	**FALSE** ☐	**I DO NOT KNOW** ☐
**22**	*Dairy are a good iron source*		
	**TRUE** ☐	**FALSE** ☐	**I DO NOT KNOW** ☐
**23**	*Carrots are a good source of vitamin A*		
	**TRUE** ☐	**FALSE** ☐	**I DO NOT KNOW** ☐

24.L'Indice Glicemico di un alimento:(indicare l'unica risposta corretta)

Indica il contenuto di carboidrati di un alimento	☐
Permette di classificare gli alimenti in base all'effetto che hanno sul livello di glucosio nel sangue (glicemia)	☐
Indica la velocità con cui aumenta la glicemia in seguito all'assunzione di un alimento contenente una quantità nota di proteine	☐
Indica la densità calorica di un alimento	☐

Are these claims true or false? (only one answer is possible)

**25**	*An unbalanced diet is the only risk factor for the development of cardiovascular disease*		
	**TRUE** ☐	**FALSE** ☐	**I DO NOT KNOW** ☐
**26**	*In the obesity the diet play an important rule, physical activity no*		
	**TRUE** ☐	**FALSE** ☐	**I DO NOT KNOW** ☐
**27**	*A low calcium and vitamin D intake during life, associated with a lack of physical activity may increase fracture risk*		
	**TRUE** ☐	**FALSE** ☐	**I DO NOT KNOW** ☐
**28**	*The fiber helps to ease constipation*		
	**TRUE** ☐	**FALSE** ☐	**I DO NOT KNOW** ☐
**29**	*To obtain an healthy weight loss the carbohydrates must not be removed from the diet*		
	**TRUE** ☐	**FALSE** ☐	**I DO NOT KNOW** ☐

SECTION 2: SPORT NUTRITION

Are these claims true or false? (only one answer is possible)

**30**	*To eat carbohydrate is no good for an athlete*		
	**TRUE** ☐	**FALSE** ☐	**I DO NOT KNOW** ☐
**31**	*B-group vitamins play an important role in muscle metabolism*		
	**TRUE** ☐	**FALSE** ☐	**I DO NOT KNOW** ☐
**32**	*Athlethes must reduce the fat intake to a minimum*		
	**TRUE** ☐	**FALSE** ☐	**I DO NOT KNOW** ☐
**33**	*Consuming carbohydrates after 5pm can enhance performance*		
	**TRUE** ☐	**FALSE** ☐	**I DO NOT KNOW** ☐
**34**	*Eating more protein will make muscles bigger*		
	**TRUE** ☐	**FALSE** ☐	**I DO NOT KNOW** ☐
**35**	*Per una persona che fa sport la dieta non deve contenere più del 15% di grassi*		
	**TRUE** ☐	**FALSE** ☐	**I DO NOT KNOW** ☐
**36**	*Athletes can eat whatever they want because they have a fast metabolism*		
	**TRUE** ☐	**FALSE** ☐	**I DO NOT KNOW** ☐
**40**	*Physical exercise is the main factor improving muscular strength*		
	**TRUE** ☐	**FALSE** ☐	**I DO NOT KNOW** ☐
**41**	*An excessive dietary protein intake can lead to liver and kidney damage*		
	**TRUE** ☐	**FALSE** ☐	**I DO NOT KNOW** ☐
**42**	*At the end of a training session an athlete must have a meal*		
	**TRUE** ☐	**FALSE** ☐	**I DO NOT KNOW** ☐
**43**	*To reduce pasta, potato and bread intake during the training period is needed*		
	**TRUE** ☐	**FALSE** ☐	**I DO NOT KNOW** ☐
**44**	*A man and woman of the same age, practicing the same sport have the same energy requirements*		
	**TRUE** ☐	**FALSE** ☐	**I DO NOT KNOW** ☐
**45**	*It is advisable for an athlete eating a low glycemic index meal but rich in carbohydrates, 1-2 hours after training*		
	**TRUE** ☐	**FALSE** ☐	**I DO NOT KNOW** ☐
**46**	*Athletes practicing extensive training have double protein requirement than the general population*		
	**TRUE** ☐	**FALSE** ☐	**I DO NOT KNOW** ☐
**47**	*Drinking fluids before, during and after a competition is needed*		
	**TRUE** ☐	**FALSE** ☐	**I DO NOT KNOW** ☐
**48**	*Coaches must not allow drinking fluid during a training*		
	**TRUE** ☐	**FALSE** ☐	**I DO NOT KNOW** ☐
**50**	*The best advice for athletes is to drink when they are thirsty*		
	**TRUE** ☐	**FALSE** ☐	**I DO NOT KNOW** ☐
**51**	*For an athlete cold water quenches thirst better*		
	**TRUE** ☐	**FALSE** ☐	**I DO NOT KNOW** ☐
**52**	*Durante l'allenamento uno sportivo può tenere in bocca un cubetto di ghiaccio per dissetarsi*		
	**TRUE** ☐	**FALSE** ☐	**I DO NOT KNOW** ☐

**53**	*Un importante aiuto per la prestazione di un atleta è l'acqua*		
	**TRUE** ☐	**FALSE** ☐	**I DO NOT KNOW** ☐
**54**	*Bevande per sportivi e bevande energetiche sono la stessa cosa*		
	**TRUE** ☐	**FALSE** ☐	**I DO NOT KNOW** ☐
**55**	*Le bevande per sportivi contengono sali minerali*		
	**TRUE** ☐	**FALSE** ☐	**I DO NOT KNOW** ☐

56.Qual è la bevanda più appropriata dopo due ore di allenamento?

Bevanda energetica	☐
Bevanda per sportivi	☐
Succo di frutta	☐
Cola	☐
Non so	☐

Are these claims true or false? (only one answer is possible)

**57**	*Gli integratori possono essere validi sostituti dei pasti per lo sportivo*		
	**TRUE** ☐	**FALSE** ☐	**I DO NOT KNOW** ☐
**58**	*Un atleta non potrà mai soddisfare il suo fabbisogno di ferro con l'alimentazione, è necessario un supplemento*		
	**TRUE** ☐	**FALSE** ☐	**I DO NOT KNOW** ☐
**59**	*Le bevande per sportivi contengono caffeina*		
	**TRUE** ☐	**FALSE** ☐	**I DO NOT KNOW** ☐
**60**	*Se un atleta non riesce a coprire il proprio fabbisogno di proteine con la sola alimentazione è necessaria un'integrazione di amminoacidi*		
	**TRUE** ☐	**FALSE** ☐	**I DO NOT KNOW** ☐
**61**	*L'eritropoietina è una sostanza dopante usata per migliorare la resistenza*		
	**TRUE** ☐	**FALSE** ☐	**I DO NOT KNOW** ☐
**62**	*Gli integratori alimentari sono sempre necessari, sia nello sport agonistico che non agonistico*		
	**TRUE** ☐	**FALSE** ☐	**I DO NOT KNOW** ☐
**63**	*Poiché gli integratori alimentari sono innocui, non è necessario il consiglio dello specialista*		
	**TRUE** ☐	**FALSE** ☐	**I DO NOT KNOW** ☐
**64**	*Il supplemento di vitamina C è sempre necessario a chi pratica sport di forza*		
	**TRUE** ☐	**FALSE** ☐	**I DO NOT KNOW** ☐
**65**	*Mangiare scondito è il miglior modo per fare massa e tonificare per uno sportivo*		
	**TRUE** ☐	**FALSE** ☐	**I DO NOT KNOW** ☐
**66**	*Carne e albume d'uovo contengono proteine, gli altri alimenti no. Questa è la base dell'alimentazione di uno sportivo*		
	**TRUE** ☐	**FALSE** ☐	**I DO NOT KNOW** ☐
**67**	*I prodotti light possono essere consumati a piacimento da uno sportivo*		
	**TRUE** ☐	**FALSE** ☐	**I DO NOT KNOW** ☐
